# Rab23 is required for cardiac progenitor cell differentiation and positively-regulates Wnt11/AP-1 signalling in zebrafish

**DOI:** 10.1186/2046-2530-1-S1-O6

**Published:** 2012-11-16

**Authors:** D Jenkins, PL Beales, AOM Wilkie

**Affiliations:** 1 UCL Institute of Child Health, London, UK; 2 Weatherall Institute of Molecular Medicine, Oxford, UK

## 

There has been considerable recent interest in the roles of vesicle transport proteins in ciliary biology and regulation of signal transduction. We have previously discovered *RAB23* mutations in Carpenter syndrome, in which patients present with craniofacial and cardiac malformations, postnatal obesity and tumour formation, and Rab23 regulates trafficking of hedgehog signalling components between cilia and the cytoplasm. In the current study we investigated whether Rab23 may also regulate other signalling pathways. Wnt signalling pathways regulate a variety of developmental processes, yet the mechanisms of Wnt signal transduction remain unclear. We show that zebrafish deficient for Rab23 or its GTPase-activating protein, Evi5l, exhibit abnormal heart formation, which we attribute to a requirement for rab23 in the differentiation of cardiac progenitor cells. We delineate a Wnt11 signalling pathway that is implicated in cardiomyocyte differentiation, which acts via phospholipase C, inositol triphosphate receptor, and protein kinase C, to regulate activator protein 1 (AP-1) transcriptional activity downstream of c-Jun N-terminal kinase. We also show that Rab23 interacts functionally with several components of this pathway to regulate transcription by AP-1. Collectively, these findings identify Rab23 as a novel positive-regulator of non-canonical Wnt11/AP-1 signalling, which may be relevant to cardiovascular disease and stem cell biology. We also present evidence that Rab23 is dispensible for hedgehog signalling in zebrafish, and we propose that this reflects previously reported evolutionary differences in the requirement for cilia in hedgehog signal transduction. Collectively, our findings suggest that Rab23 may integrate multiple signalling pathways, possibly playing both cilia-dependent and –independent roles.

